# Radiation exposure levels within timber industries in Calabar, Nigeria

**DOI:** 10.4103/0971-6203.51937

**Published:** 2009

**Authors:** S. O. Inyang, I. S. Inyang, N. O. Egbe

**Affiliations:** Departments of Physics, University of Calabar, Calabar - 540001, Cross River State, Nigeria; 1 Department of Curriculum and Teaching, University of Calabar, Calabar - 540001, Cross River State, Nigeria; 2 Department of Radiography, University of Calabar, Calabar - 540001, Cross River State, Nigeria

**Keywords:** Occupational exposure, radionuclide, regulatory control, timber

## Abstract

The UNSCEAR (2000) observed that there could be some exposure at work which would require regulatory control but is not really considered. This study was, therefore, set up to evaluate the effective dose in timber industries in Calabar, Nigeria to determine if the evaluated dose levels could lead to any radiological health effect in the workers, and also determine if the industries require regulatory control. The gamma ray exposure at four timber industries measured using an exposure meter were converted to effective dose and compared with the public and occupational values. The evaluated effective dose values in the timber industries were below public and occupational exposure limits and may not necessarily result in any radiological health hazard. Therefore, they may not require regulatory control.

## Introduction

Ionizing radiation and radioactivity are found naturally within the environment and their levels depend generally on the distribution of natural radionuclides within the environment. Human activities involving mining, the use and processing of radionuclides or items which contain radionuclides, can enhance the levels of environmental radiation.

Industrial and medical uses of radiation are beneficial to man. The benefits derived in these cases notwithstanding, use of radiation and radionuclides can be hazardous to man and the environment if such use is not regulated; and exposure to radiation kept within acceptable limits. Evidence of radiation damage such as radiation induced malignancies and damage to genetic material have been observed from long term epidemiological studies of population exposed to radiation.[1]

The mandate to control nuclear and radiation generating sources in Nigeria is vested with the Nigerian Nuclear Regulatory Authority which is authorized by law to ensure that radiation protection and safety regulations are adhered to. Several studies have been conducted in Nigeria to assess the environmental radiation and radioactivity levels within most part of the country. Among the recent ones are dose measurements along the creeks of Calabar River,[2] dose assessment within the mines in Benue State,[3] radioactivity in roots,[4] outdoor radiation level[5–8] and radioactivity in building.[9–11] More data on radioactivity and radiation levels in Nigeria are also available.[12–16]

Occupational exposure involves every individual exposure to ionizing radiation at place of work resulting directly from work. Occupational exposures are observed in operations involving uranium mining and milling, nuclear fuel fabrication, radiation medicine, accelerator operation, civilian aviation, mineral mining, defence nuclear activities, and educational and research activities. These operations stated here notwithstanding, UNSCEAR[17] observed that there is a significant exposure of workers to long-lived natural sources of radionuclides coming from dust during processing and handling of bulk quantities of minerals and other material which may require regulatory control to monitor and record occupational exposures at such operation sites. They further maintain that there is less awareness of workers exposures from enhanced levels of natural radiation in other settings which could require regulatory control.

Timber used for furniture, roofing and fibre for pulp and paper[18] in Nigeria is usually procured from the forests. The timber business, which involves processing of logs into plywood, storage of processed products and sales of logs for roofing and carpentry, employs several people who earn their living on it. The timber used in Calabar, the Cross River State capital, is derived from forests in the Northern part of the State. High levels of uranium and thorium have been observed in the soils of some of the forests where timber is sourced to Calabar.[19–22]

Plant is known as one of the paths through which radioactivity and radiation get to man.[23] This study aims at preliminary determination if the timber in Calabar, obtained from the forests mentioned above could retain some radioactivity which could raise the radiation level within the timber industries in Calabar. The study is also aimed at deducing if the evaluated effective dose levels within the timber industries in Calabar could result in any radiological health effect to the workers in the industries. The study was conducted by measuring the gamma ray exposure levels within timber industries (one factory and three markets) in Calabar using Ludlum exposure meter (model 12 SA) from Ludlum Measurements Inc., USA.

## Materials and Methods

**Figure 1:**
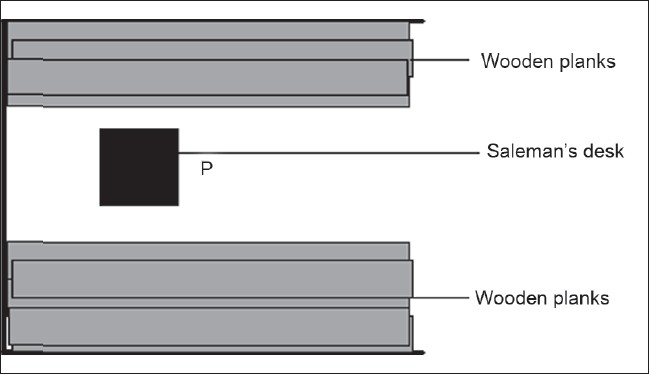
Typical floor arrangement of timber markets' sales outlet

One timber factory and three timber markets which constitute all the timber industries located within Calabar were selected for the study. The factory converts timber procured from forests in the northern part of Cross River State, Nigeria into plywood and planks of different sizes. The factory consists of administrative block, processing room and stores for finished products and fresh timber being brought in from the forests. The timber markets consist of opened stalls which are used as sales outlets or machine rooms. The machine rooms are used for re-sizing the timber to different sizes before placing them in the sales outlets for sale to buyers. Figure 1 gives an example of the layout of a typical sales outlet where the planks are piled up from the floor to the roof of the shade.

A radiation survey was conducted using an exposure meter (model 12 SA) from Ludlum Measurements Inc., USA with calibration traceable to the National Institute of Standards and Technology (NIST). The meter was calibrated in unit of *μ*R/h, with error within  ±10%, using a Cs – 137 gamma source. Though other dosimeters such as thermoluminescence dosimeter (TLD) and sodium iodide (NaI) spectroscopy could be used for this study, the Ludlum exposure meter was selected based on availability and because the study is meant to be a preliminary investigation whose result, as a guide, would help in deciding if the level of radiation exposure in the wood industry requires regulatory control. Gamma ray exposure within the factory was taken at points where workers usually stay within the fresh timber store, finished product store, processing room and administrative block. During the measurement, the exposure meter was placed at a central point P [Figure 1] of each store in a manner that the entrance window of the meter points directly towards a pile of timber. The measurement was repeated at least 15 times in each store with the meter pointing at the piles of timber within the store in different directions.

The measurement process was repeated for the sales outlets and machine rooms within the timber markets selected. To establish the average background not related to the processing and handling of timber, measurement of gamma ray exposure was taken at least 10 m away from any of the known timber industries.[13] All measurements of exposure in this work were taken between the hours of 13 and 16 during which maximum instrument response to environmental radiation exposures within Calabar have been observed.[5]

Radiological impact of radiation exposure measurement is usually determined through the use of the whole body effective dose measured in seivert (Sv) or its subunits. In estimating the effective dose, the exposure measurements taken in the units of *μ*R/h were first multiplying by 76 *μ*Gy/h/*μ*R/h to convert them to absorbed dose (not shown). The absorbed dose values (not shown) were further multiplied with 0.7 *μ*Sv/yr/ *μ*Gy/h to convert to effective dose in *μ*Sv/yr.[23]

## Results

Gamma ray exposure values measured in *μ*R/h with the corresponding maximum standard deviation (SD) and the associated effective doses in mSv/yr estimated from the measured gamma ray exposure values are given in Table 1. The exposure values reported in Table 1 are average values of at least 15 measurements taken under similar conditions and environment. In essence, exposure measurements taken at each timber store, was summed up and the average value recorded. Conversion of gamma ray exposure values to absorbed doses (values not shown), and subsequently from absorbed doses to effective doses follow the method discussed in NCRP[23] and is briefly explained above under material and methods.

**Table 1:** Radiation exposure measurement in timber industries

*Timber industry*	*Site number*	*Exposure ± (SD) μR/hr*	*Effective Dose mSv/yr*
Timber factory			
Outside space	1	11.02 ± 0.63	0.586
Waste dump	2	13.23 ± 0.78	0.704
Timber store	3	14.86 ± 0.73	0.791
Processing room	4	14.57 ± 0.64	0.775
Product store	5	13.90 ± 0.54	0.739
Admin block	6	10.95 ± 0.85	0.583
Timber market A			
Outside space	1	13.05 ± 0.37	0.694
Waste dump	2	13.19 ± 0.78	0.702
Timber store	3	13.28 ± 0.89	0.744
Machine room	4	14.76 ± 0.79	0.785
Product store	5	14.12 ± 0.57	0.751
Timber market B			
Outside space	1	12.96 ± 0.73	0.689
Waste dump	2	13.16 ± 0.80	0.700
Timber store	3	13.28 ± 0.73	0.729
Machine room	4	14.76 ± 0.93	0.778
Product store	5	13.91 ± 0.75	0.740
Timber market C			
Outside space	1	13.36 ± 0.91	0.711
Waste dump	2	13.25 ± 0.65	0.705
Timber store	3	13.28 ± 0.97	0.735
Machine room	4	14.57 ± 0.74	0.775
Product store	5	13.63 ± 0.76	0.725
Environmental background		10.37 ± 0.58	0.552

The exposure values and effective doses ranged between 10.95 – 14.86 *μ*R/hr and 0.583 – 0.891 mSv/yr respectively in timber industries. The average value of background gamma ray exposure was 10.73 *μ*R/hr, with a corresponding whole body effective dose of 0.552 mSv/yr; dose levels within the different timber industries were not significantly different.

## Discussion

A survey of radiation exposure at work is intended to evaluate whether the levels of exposure are sufficiently high to the extent that radiological health effect may result and such places may require the implementation of regulatory control. Effective doses estimated for all the studied timber industries were higher than the average environmental background value. This may be attributed to residual radioactivity in the timber which could have accumulated within the timber from the soil of the forests. The estimated values of effective dose within the timber industries were lower than the average value of 0.115 *μ*Sv/yr (1.01 mSv/yr) obtained by Uwah and Akpan[2] for the environs of Calabar River. This higher dose value within the environs of Calabar River is most likely due to the deposition of particles, which may include some radionuclides, along the river bank by the flowing water.

**Figure 2:**
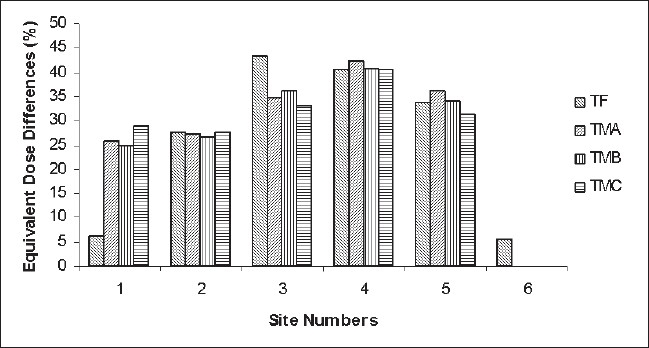
Equivalent Dose Difference (%) for the measurement sites in the timber factory (TF), timber market A (TMA), timber market B (TMB) and timber market C (TMC)

Figure 2 and Table 1 show that most of the dose values at the different measurement sites within the timber factory were higher than the corresponding values at the timber markets. These differences could be attributed to the differences in the structures housing the factory and markets. The markets have open stalls with roof supported by pillars, while the factory had roof over brick walls with large windows for ventilation located within the walls. The building style in the markets offer better ventilation than the factory and reduced the radiation build-up within the stalls. The higher values observed within the factory may be the result of brick masonry used in the factory construction. An average dose value of 0.583 mSv/yr obtained within the administrative block (no processing or storing of timber) of the factory, which gives a difference of 5.62% compared to the environmental background value [Figure 2] further confirms that the building brick could be the source of the higher dose value within the administrative block. The situation here is similar to those reported by Arabi *et al*[24] and UNSCEAR.[17]

A percentage difference of 6.16% in effective dose value was obtained in the outside space of the timber factory while values ranging between 24.50% and 29.00% were obtained for the timber markets. The observed differences may be due to the impact of distance from the radiation source on the exposure obtained at a point. The outside space within the factory is larger than those found in the markets, and exist between the administrative block and the processing room/product stores. Those in the markets exist between two lines of stalls and are not as large as the one in the factory.

The evaluated effective doses within the timber industries were lower than 1 mSv/yr which is the public exposure limit and as well lower than the occupational exposure limit of 20 mSv/yr;[25] and therefore may not attract regulatory controls.

## Conclusion

The effective doses obtained within the investigated timber industries, although higher than the environmental background, are not sufficiently high to warrant regulatory control and may not cause any radiological health hazard in workers within the industries. However, further investigation is necessary to establish the types and concentrations of the radionuclides present in the timber, and relate same to the values obtained from soils of the forest of which the timbers were sourced.
